# Exertional rhabdomyolysis in female amateur triathletes

**DOI:** 10.1186/cc11066

**Published:** 2012-03-20

**Authors:** V Meighan

**Affiliations:** 1 Galway University Hospital, Galway, Ireland

## Introduction

Multisport endurance events are becoming increasingly popular in Ireland. Overexertion, especially in the heat, of overweight or poorly conditioned athletes increases the risk of rhabdomyolysis. This study presents a case series of three female amateur triathletes presenting with acute abdominal pain caused by rhabdomyolysis.

## Methods

The medical case notes of three female athletes presenting to the emergency department were reviewed.

## Results

All three patients presented with abdominal pain after triathlon training. On admission, creatinine kinase levels were over 30,000 in all three cases and all required acute hospital admission for pain relief and intravenous fluids to prevent renal failure.

## Conclusion

Exertional rhabdomyolysis is not rare, but rarely do such patients present to the emergency department with acute abdominal pain. Whilst triathlon training is popular among amateur sports people, awareness must be raised to train appropriately under proper conditions.

